# Supplementary material for: Investigating the influence of physiologically relevant hydrostatic pressure on CHO cell batch culture
Source: Sci Rep. 2021 Jan 8;11:162. doi: 10.1038/s41598-020-80576-8 (PMC7794228; doi:10.1038/s41598-020-80576-8)
Supplement: Supplementary file 1 — Supplementary Information 1. [file 41598_2020_80576_MOESM1_ESM.zip › S1/IgG/Feb14 0 30 60 90/90mmHg Feb10 2.pdf]

Data File C:\Users\P...mStation\1\Data\TK_CHO_IgG 2019-02-14 14-17-55\042-10-ML 90 Feb10.D
Sample Name: ML 90 Feb10

===

```
Acq. Operator   : SYSTEM                        Seq. Line :  42
Sample Operator : SYSTEM
Acq. Instrument : HPLC-0039                      Location :   10
Injection Date  : 2/14/2019 5:15:18 PM                Inj :   1
                                              Inj Volume : 100.000 µl
Method          : C:\Users\Public\Documents\ChemStation\1\Data\TK_CHO_IgG 2019-02-14 14-17-55
                  \CHO_IgG_LC.M (Sequence Method)
Last changed    : 2/8/2019 3:17:23 PM by SYSTEM
```

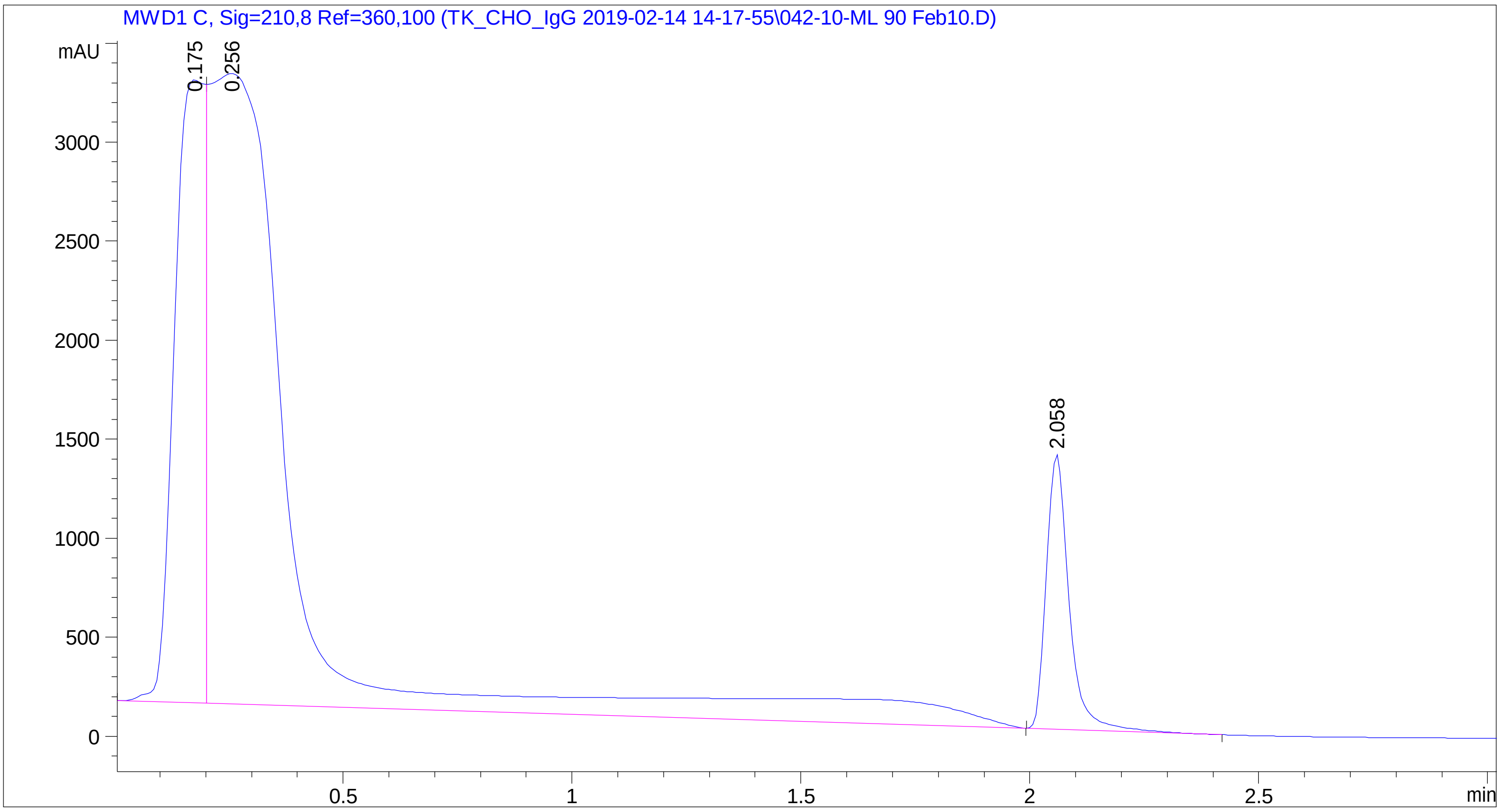

===

Area Percent Report

===

```
Sorted By             :      Signal
Multiplier            :      1.0000
Dilution              :      1.0000
Use Multiplier & Dilution Factor with ISTDs
```

Signal 1: MWD1 C, Sig=210,8 Ref=360,100

Peak #	RetTime [min]	Type	Width [min]	Area [mAU*s]	Height [mAU]	Area %
1	0.175	BV	0.0685	1.41557e4	3146.60181	23.7752
2	0.256	VB	0.1846	4.05538e4	3183.54614	68.1122
3	2.058	BBA	0.0526	4830.15869	1388.65747	8.1125
Totals :				5.95396e4	7718.80542	

===

*** End of Report ***
